# Spontaneous intermuscular hematoma in mixed connective tissue disease: a case report

**DOI:** 10.3389/fsurg.2026.1719697

**Published:** 2026-08-19

**Authors:** Zhaohua Pei, Yun Chen, Linjun Zhao, Long Zheng, Yuanhan Lin, Xue Zhao

**Affiliations:** 1 Department of Emergency Medicine, Zhejiang Chinese Medical University, Hangzhou, Zhejiang Province, China; 2 Department of Emergency Medicine, The First People's Hospital of Hangzhou Lin'an District, Hangzhou, Zhejiang Province, China; 3 Department of General Practice, The First People's Hospital of Hangzhou Lin'an District, Hangzhou, Zhejiang Province, China; 4 Department of General Practice, Zhejiang Chinese Medical University, Hangzhou, Zhejiang Province, China; 5 Department of Emergency Medicine, Affliated Hangzhou First People's Hospital, School of Medicine, Westlake University, Hangzhou, Zhejiang Province, China

**Keywords:** case report, intervene, mixed connective tissue disease, spontaneous intermuscular hematoma, transcatheter arterial embolization

## Abstract

Spontaneous large intermuscular hematoma secondary to mixed connective tissue disease (MCTD) is extremely rare. We report a 57-year-old female with long-standing MCTD admitted for abdominal pain and fever (septic shock due to E. coli pyelonephritis). On hospital day 8, she developed a left thigh hematoma (10  ×  14 cm). CTA failed to identify the bleeding vessel, but transcatheter arterial embolization (TAE) stabilized the hematoma. Subsequent surgical debridement was performed for hematoma-induced tissue necrosis. This case highlights that MCTD-related vascular fragility, combined with sepsis and DIC, increases hematoma risk. Prompt TAE and surgical intervention are critical to prevent catastrophic outcomes.

## Introduction

Mixed connective tissue disease (MCTD) is an autoimmune disorder characterized by two key features: 1) concurrent or sequential involvement of at least two connective tissue diseases (e.g., systemic lupus erythematosus (SLE), systemic sclerosis (SSc), polymyositis (PM), rheumatoid arthritis (RA) (1, 2); 2) presence of anti-U1 ribonucleoprotein (RNP) antibodies and Raynaud's phenomenon (3, 4). In MCTD, vascular injury and sclerosis increase the risk of hemorrhagic complications- this is particularly relevant for rare severe bleeding events, such as spontaneous intermuscular hematoma. We herein present a case of a spontaneous large intermuscular hematoma in the lower limb of a patient with MCTD and review current research on its pathophysiological mechanisms. Compared to intermuscular hematomas induced by anticoagulant therapy, those associated with MCTD tend to have more severe consequences (e.g., rapid progression, tissue necrosis, as observed in this case). Therefore, early recognition of spontaneous hematomas and prompt intervention are crucial to preventing catastrophic outcomes in patients with MCTD.

The serological hallmark of MCTD is high-titer autoantibodies against U1-ribonucleoprotein (U1-RNP) (5, 6). Anti-U1-RNPantibodies activate monocytes and autoreactive CD4+ T cells, leading to the upregulation of interleukin (IL)-1 and IL-6, key mediators of autoimmune responses that induce intercellular adhesion molecule-1 (ICAM-1) and endothelial leukocyte adhesion molecule-1 (ELAM-1). These molecules collectively promote endothelial activation and injury (7, 8). Vascular pathology in MCTD often initially manifests as Raynaud's syndrome, characterized by pericyte over-expression and eventual intimal fibrosis and occlusion (9–11). Chronic inflammation, endothelial dysfunction, and elevated pro-inflammatory cytokines in MCTD increase atherosclerosis risk and further exacerbate vascular fragility (12). Autoantibodies or complement deposition in autoimmune diseases also enhance platelet activation (13), and scleroderma-like patients show increased platelet activation that contributes to vascular complications (14). Notably, excessive platelet activation results in the release of vasoactive substances, causing intense vasoconstriction and subsequent hemorrhage.

Spontaneous soft-tissue and intermuscular hematomas are uncommon, occurring predominantly in patients with coagulopathies, anticoagulant use, or connective tissue diseases. In autoimmune conditions, the pathogenesis is often multifactorial, involving corticosteroid use, vasculopathy, thrombocytopenia, or acquired coagulation defects. Such hematomas have been reported in systemic lupus erythematosus and rheumatoid arthritis, but remain exceedingly rare in mixed connective tissue disease (MCTD). Management requires a multidisciplinary approach, including coagulopathy correction, CTA for bleeding localization, angiographic embolization for active extravasation, and surgical intervention when necrosis or compartment syndrome develops. Herein, we present a rare case of spontaneous large intermuscular hematoma of the lower extremity in a patient with MCTD.

## Case report

### Patient presentation and admission (Day 1)

A 57-year-old female was admitted with a one-week history of fever that had acutely worsened over the preceding 24 h. Her past medical history was significant for hypertension (>15 years), MCTD (>10 years) managed with long-term prednisone acetate 5 mg daily, and interstitial lung disease (>1 year) treated with mycophenolate mofetil dispersible tablets 0.5 g twice daily (total 1 g/day).

On Day 1 (November 9, 2024), the patient presented with a critical condition. Vital signs were as follows: temperature 37.9 °C, pulse 112 beats/min (tachycardia), respiratory rate 24 breaths/min (tachypnea), blood pressure 94/49 mmHg (meeting criteria for septic shock hypotension), and oxygen saturation 97%. Laboratory studies immediately revealed tissue hypoperfusion and severe infection: lactate 4.86 mmol/L, procalcitonin (PCT) > 100 ng/mL, and platelets (PLT) 56  ×  10⁹/L. Central venous pressure (CVP) measured 14 cmH₂O after catheterization, and 24-hour urine output was 700 mL. Collectively, these findings—hypotension, tissue hypoperfusion, and evidence of severe infection—confirmed the diagnosis of septic shock.

### Initial management and immunosuppressive adjustment (Day 1)

Urgent hemodynamic support was initiated, consisting of aggressive fluid resuscitation (3,071 mL of crystalloids and colloids within the first 24 h) and norepinephrine infusion (initial concentration 4 mg/50 mL, estimated at ∼0.08 μg/kg/min, titrated to maintain mean arterial pressure >65 mmHg). Concurrently, the immunosuppressive regimen was modified to balance MCTD control with infection management: mycophenolate mofetil was completely withheld on Day 3 (November 11), and prednisone was converted to intravenous methylprednisolone 40 mg daily for 7 days, followed by tapering to 20 mg daily, then transitioned back to oral prednisone 10 mg daily at discharge.

Physical examination on admission revealed drowsiness (consistent with hypoperfusion-induced altered mental status), clear bilateral lung sounds without rales (excluding primary pulmonary infection), a soft abdomen with mild epigastric tenderness but no rebound or guarding, and scattered purpuric and ecchymotic lesions on both lower limbs (consistent with the patient's known MCTD-related vasculopathy).

### Auxiliary investigations (Day 1)

Admission laboratory tests demonstrated leukocytosis, severe anemia, thrombocytopenia, and marked coagulopathy, consistent with septic shock and evolving DIC. Key results are summarized in Table 1. Autoantibody profiling was positive for anti-nuclear antibody (titer 1:1000), anti-U1-RNP (99), and anti-Ro-52 (86). Chest computed tomography (CT) revealed bilateral interstitial inflammatory changes and localized pleural thickening. Lower-extremity arterial and venous ultrasound showed normal blood flow, effectively ruling out vascular infection.

**Table 1:** Key laboratory findings on admission (November 9–10, 2024).

Parameter	Value	Reference range
White blood cell	25.2 × 10⁹/L	4–10 × 10⁹/L
Hemoglobin	75 g/L	115–150 g/L
Platelets	56 × 10⁹/L	100–300 × 10⁹/L
Procalcitonin	>100 ng/mL	<0.05 ng/mL
C-reactive protein	283.9 mg/L	<5 mg/L
Lactate	4.86 mmol/L	<2 mmol/L
Prothrombin time	16.3 s	11–14 s
International normalized ratio	1.45	0.8–1.2
Activated Partial Thromboplastin Time	57.3 s	25–40 s
Thrombin Time	38.2 s	16–18 s
D-dimer	99,360 μg/L FEU	<500 μg/L FEU
Fibrinogen	3.83 g/L	2–4 g/L

### Microbiological diagnosis and DIC confirmation (days 2–5)

On Day 2 (November 10), follow-up laboratory tests confirmed overt disseminated intravascular coagulation (DIC), with prothrombin time 16.3 s, international normalized ratio 1.45, partial thromboplastin time 57.3 s, D-dimer 99,360 μg/L FEU, and persistent thrombocytopenia (PLT 56  ×  10⁹/L). These coagulation abnormalities significantly increased the patient's bleeding risk, predisposing her to subsequent hematoma formation.

To identify the septic focus, blood next-generation sequencing (NGS) was sent on Day 4 (November 12), and urine culture was obtained concurrently. Both subsequently detected Escherichia coli. Together with the patient's known polycystic kidney disease (noted on abdominal CT) and urinary findings (urinalysis showing occult blood +++ and protein ++), these results confirmed the diagnosis of acute pyelonephritis. Additionally, blood cultures collected on Day 5 (November 13) grew gram-negative bacilli, morphologically and biochemically consistent with E. coli, further supporting the diagnosis of E. coli bloodstream infection secondary to pyelonephritis.

### Clinical course and response to treatment (days 1–7)

Following the initiation of hemodynamic support, anti-infection therapy, and inflammatory response modulation, the patient's symptoms showed gradual improvement. Blood pressure stabilized, and laboratory parameters were closely monitored throughout this period.

### Spontaneous hematoma onset (Day 8)

**Figure 1:**
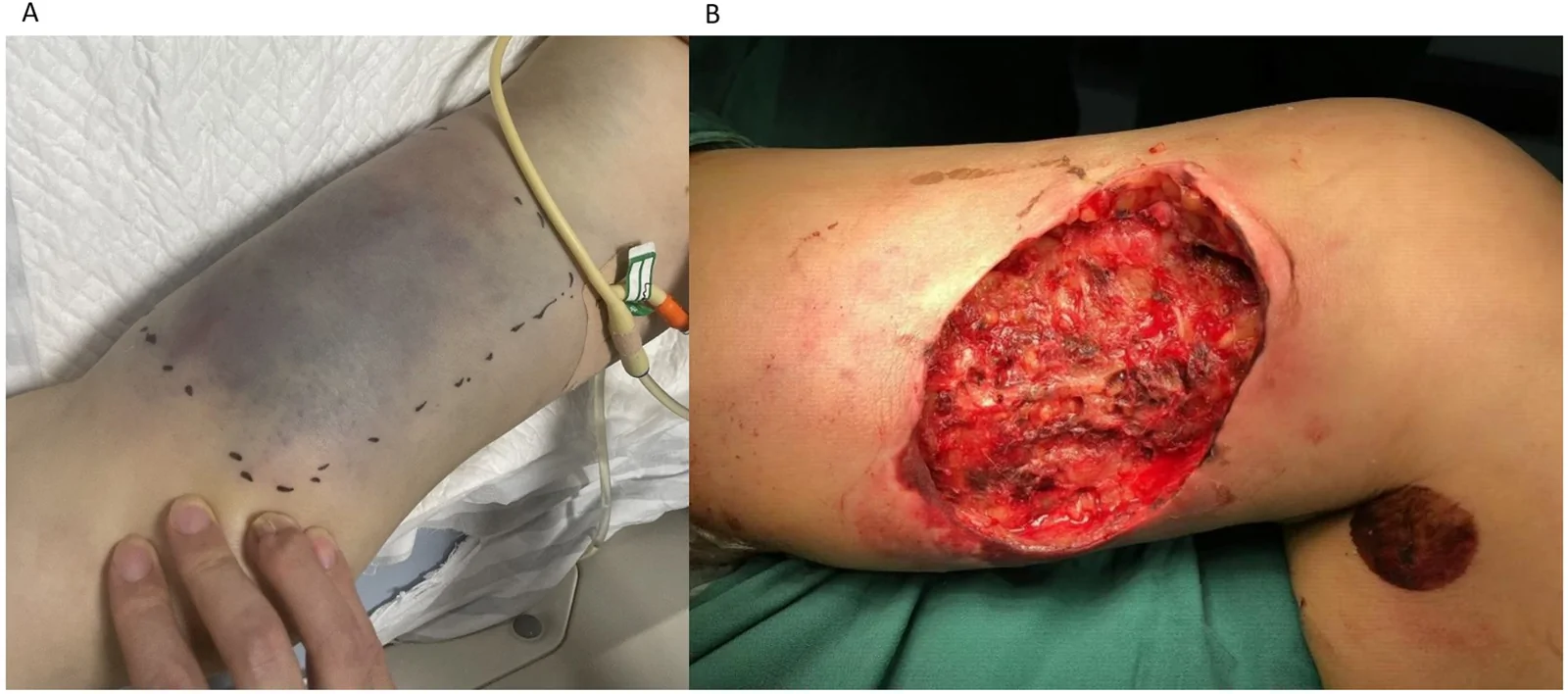
**(A)** initial hematoma; **(B)** after the hematoma is removed.

**Figure 2:**
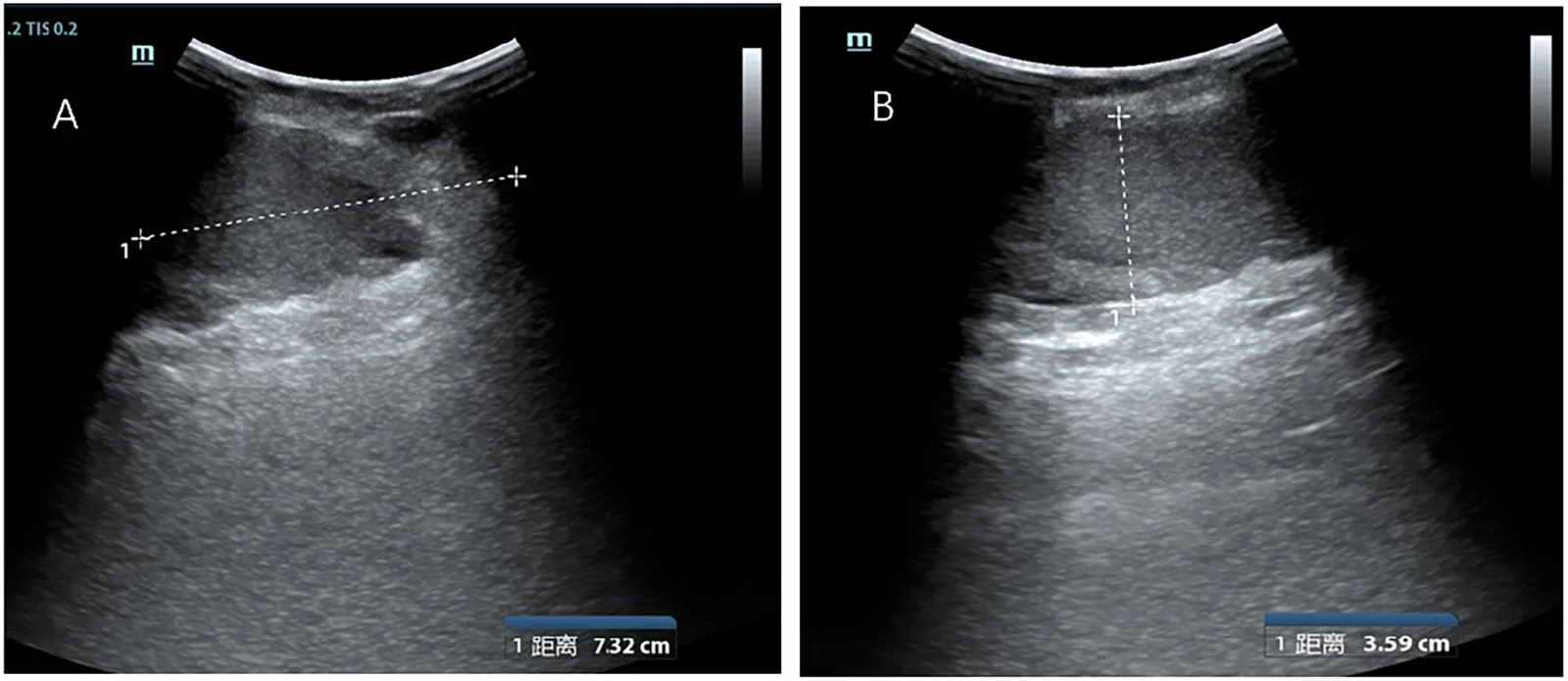
Initial sonogram of hematoma.

**Figure 3:**
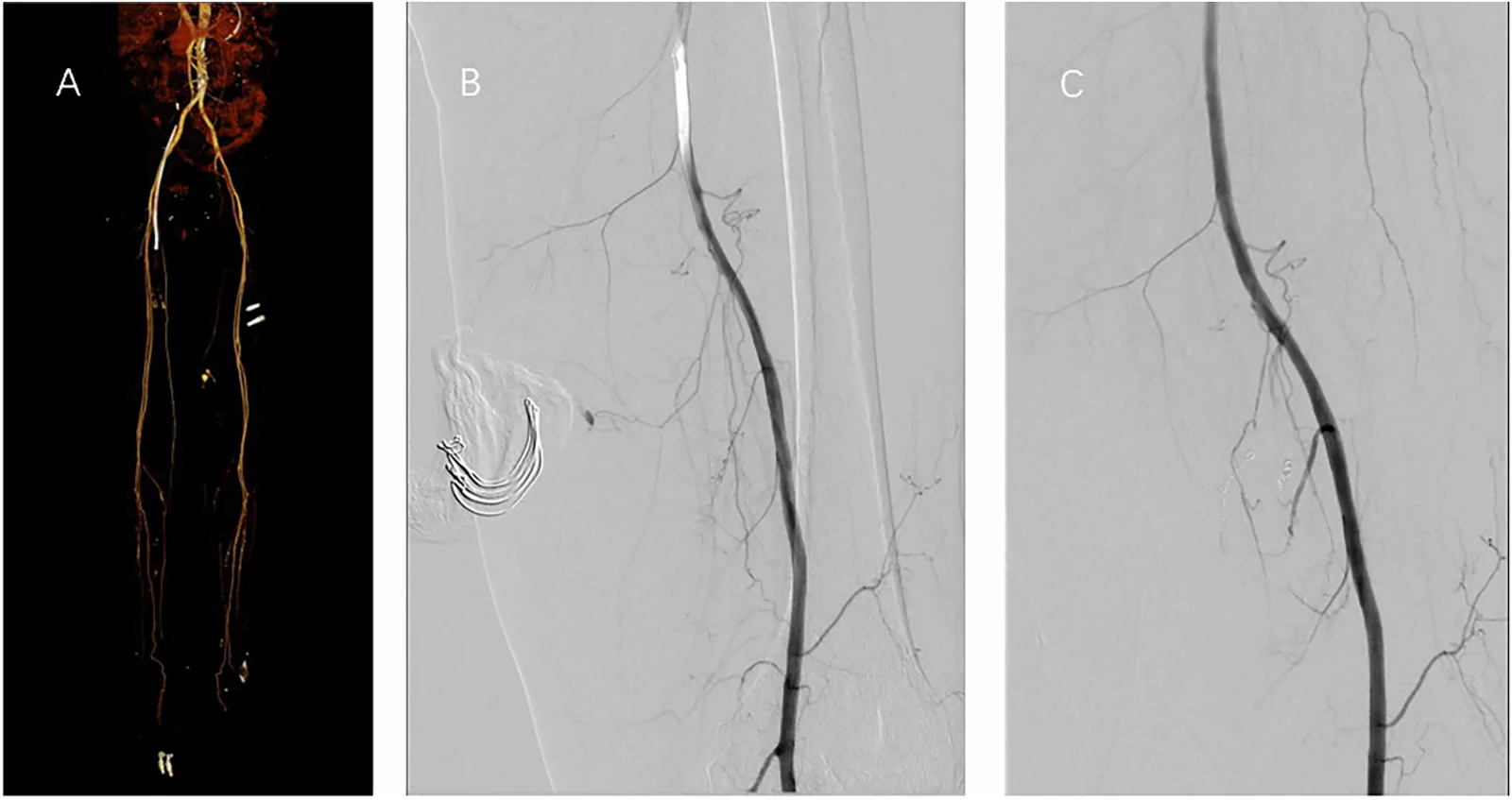
**(A)** Angiography of the lower extremities; **(B)** Contrast extravasation map; **(C)** Contrast embolization diagram.

On Day 8 (November 16, 2024), which was 6 days after DIC diagnosis and the 8th day of hospitalization, the patient developed a spontaneous bluish-black mass on the medial aspect of the left thigh, without any history of trauma or anticoagulant therapy (Figure 1A). The lesion measured approximately 10  ×  14 cm, was firm and immobile, and exhibited mildly elevated local temperature compared with the surrounding skin. Notably, the patient reported only minimal pain. Emergency ultrasound confirmed the presence of a hematoma (Figures 2A,B). Subsequent CT angiography (CTA) of the lower limb revealed a subcutaneous hematoma with exudation and active bleeding in the left medial thigh; however, no definitive arterial contrast extravasation was identified (Figure 3A). Emergency DSA clearly revealed active contrast extravasation from a muscular descending branch of the profunda femoris artery. Superselective catheterization was achieved, and embolization was performed with microcoils plus gelatin sponge particles. Post-embolization DSA confirmed technical success, showing complete cessation of extravasation, no antegrade flow in the embolized branch, and maintained patency of the main femoral and popliteal arteries. Segmental hypoperfusion of the adjacent great saphenous vein was also observed. Laboratory tests at this time showed WBC 18.8  ×  10⁹/L, RBC 2.46  ×  1,012/L, hemoglobin 66 g/L, PLT 180  ×  10⁹/L, and normalized coagulation parameters. The occurrence of a spontaneous hematoma in this clinical context was highly unusual and warranted prompt intervention.

### Diagnostic considerations and intervention (Day 8)

Given the patient's underlying MCTD (associated with vascular fragility), recent sepsis, DIC, and thrombocytopenia, the extensive intermuscular hematoma was attributed to a combination of spontaneous vascular fragility and acute sepsis-induced coagulopathy. Due to the rapid progression of the lesion and the indeterminate bleeding source on CTA, emergent arteriography of the left lower extremity was performed on the same day (Day 8). This identified active bleeding from a branch of the superficial femoral artery, which was subsequently treated with coil embolization (Figures 3B,C).

### Post-Embolization course (days 8–18)

**Figure 4:**
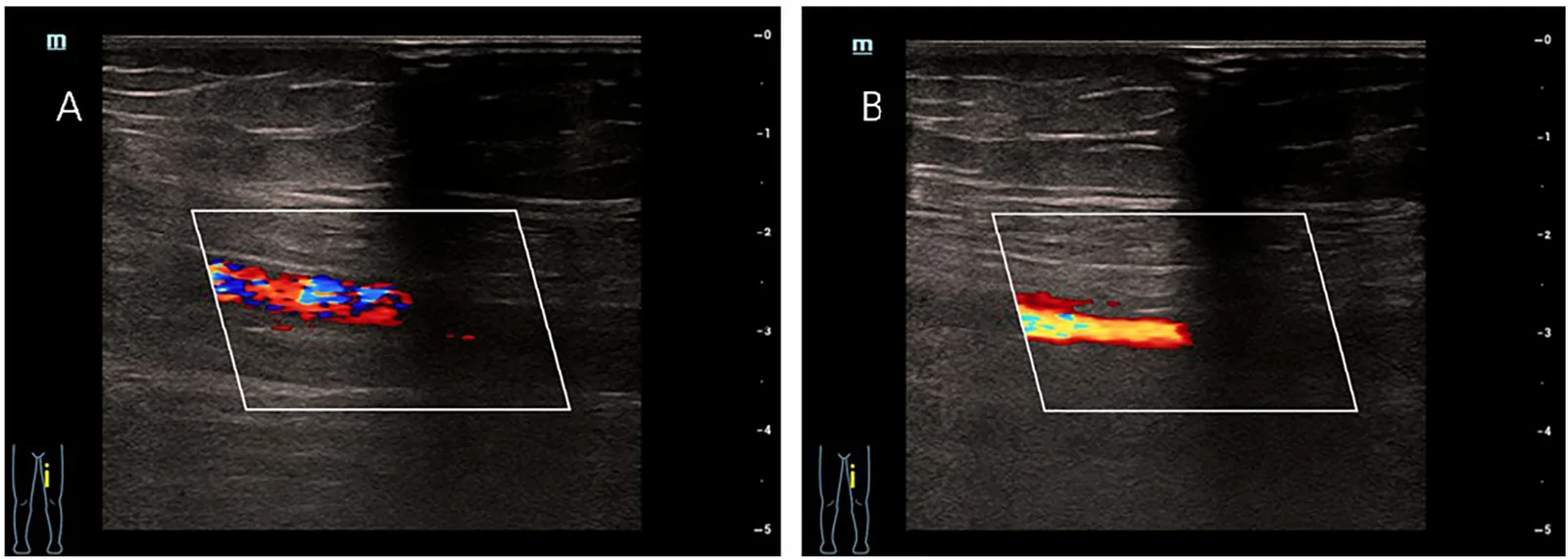
Repeat sonogram of hematoma.

Post-procedural ultrasound confirmed that the hematoma size had stabilized without further expansion, and the veins in both lower limbs remained patent (Figures 4A,B). Hemoglobin and platelet levels stabilized, and the hematoma texture progressively softened. The patient remained under close observation.

### Tissue necrosis and surgical debridement (Day 19)

However, on postoperative day 11, which corresponded to Day 19 of hospitalization, ischemic necrosis developed within the hematoma bed, with progressive skin discoloration and local tissue compromise. Despite a trial of conservative management, the necrotic changes continued to advance. Surgical debridement was therefore successfully performed on Day 19 (Figure 1B). Limb salvage was achieved, and no amputation was required. The patient subsequently recovered without further complications and was discharged in stable condition.

## Discussion

Intermuscular hematomas are clinically classified as either spontaneous or traumatic. Spontaneous hematomas are associated with bleeding disorders, tumors and arterial disease (15). Patients with MCTD exhibit vascular endothelial dysfunction and impaired endothelial progenitor cell (EPC) activity (16). Previous studies have reported MCTD patients with lupus-like manifestations developing subdural hematomas, epidural hematomas, and spontaneous perirenal hematomas (3, 17, 18); however, no reported cases of spontaneous massive intermuscular hematomas exist. It is noteworthy that intermuscular hematomas are more commonly observed in patients with hemophilia or those undergoing anticoagulant therapy (19, 20). To the best of our knowledge, this is the first reported case of a spontaneous large intermuscular hematoma in the lower extremity of a patient with MCTD, though the literature on this topic remains limited. The unique aspect of this case lies in its multifactorial etiology. While MCTD-related vasculopathy may have increased vascular fragility, concurrent factors—including sepsis, disseminated intravascular coagulation, thrombocytopenia, anemia, and immunosuppressive therapy—likely compounded the hemostatic impairment. Therefore, rather than attributing the hematoma solely to MCTD, we consider it a synergistic consequence of the patient's autoimmune diathesis and acute derangements in coagulation and inflammatory status.

While the above mechanisms explain chronic vascular vulnerability in MCTD, they do not fully account for the acute and massive nature of the hematoma in this patient. Multifactorial Pathogenesis of Spontaneous Hematoma in MCTD with Sepsis: In this patient the spontaneous large intermuscular hematoma resulted from a triad of synergistic insults: chronic endothelial injury and vascular fragility inherent to MCTD, mediated by anti-U1-RNP-driven inflammation, intimal fibrosis, and platelet hyper-activation (21, 22); acute sepsis-induced endothelial dysfunction, systemic inflammation, and thrombo-inflammation secondary to E. coli pyelonephritis (23–25); and overt DIC with consumption of platelets and clotting factors (26, 27). Together these created a “perfect storm” of vascular vulnerability, impaired hemostasis, and permissive bleeding diathesis. The normal coagulation profile on admission was misleading; rapid consumption during DIC progression precipitated hematoma formation on hospital day 8 (28). This case underscores the need to integrate autoimmune vasculopathy, septic endothelial injury, and dynamic coagulation status when assessing bleeding risk in MCTD patients with severe infection.

Clinically, these hematomas are marked by localized severe pain, swift progression, and significant local sequelae, as observed in the present case. The management of spontaneous massive intermuscular hematoma in MCTD poses significant challenges, largely due to its vascular etiology. Traditional conservative therapies, such as hemostatic agents and local compression, have often proven ineffective, and rapid progression leads to adverse outcomes. Terumi et al. reported an MCTD patient who died of massive alveolar hemorrhage with concurrent hematoma within an extremely short timeframe (29), highlighting the difficulty in locating and controlling the bleeding source. Furthermore, immunosuppressive management of MCTD must be carefully balanced against infection control. In our patient, severe sepsis secondary to E. coli pyelonephritis (confirmed by blood NGS and urine culture on November 12) prompted complete withdrawal of mycophenolate mofetil on November 11 and switch to low-dose intravenous methylprednisolone (40 mg daily) for focused anti-inflammation while avoiding excessive immunosuppression.

Contemporary therapeutic options for massive intermuscular hematoma include compression bandaging, tranexamic acid, vitamin K, blood products, TAE, and surgical evacuation (30). Because conservative measures are usually ineffective in MCTD-related hematomas, TAE is often the first-line intervention. Although ultrasound defines size and location, CTA is required to identify the responsible vessel; however, CTA may miss active extravasation when bleeding is intermittent. Thus TAE serves both diagnostic and therapeutic purposes. Surgical debridement is indicated when neurovascular compression or skin ischemia occurs (18). In our patient, CTA failed to demonstrate active extravasation, yet urgent TAE achieved hemostasis. Subsequent necrosis of skin, fascia, and muscle necessitated surgical debridement on post-embolization day 11 and again on day 24.

Several limitations should be acknowledged. First, this is a single case report, and the findings may not be generalizable. Second, and more importantly, coagulation parameters—including platelet count, PT/INR, aPTT, fibrinogen, and D-dimer—were not serially monitored at predefined intervals during the hospital course. As a result, the temporal evolution of DIC and its precise relationship to hematoma formation could not be fully characterized. This limits our ability to definitively establish DIC as the central causative mechanism, although the available data and clinical context remain supportive of this interpretation. Third, because the initial CT did not include the lower limbs, the exact time of hematoma onset could not be determined with certainty. Prospective studies or future case series with standardized coagulation monitoring and comprehensive imaging protocols are needed to further clarify these issues.

In summary, the patient presented with longstanding MCTD complicated by interstitial lung disease and livedo reticularis. Despite initially normal coagulation profiles, MCTD-related vasculopathy constituted the primary substrate, with septic shock acting as the precipitant. Sepsis-induced endothelial dysfunction, coagulopathy, and thrombocytopenia converged to trigger rapid hematoma expansion (31). Urgent TAE occluded the culprit arteriole and prevented further bleeding, but the established hematoma produced neurovascular compression and soft-tissue ischemia evidenced by tension blisters. Failure of conservative measures led to surgical evacuation and repeat debridement; the patient is currently undergoing wound care.

Spontaneous massive intermuscular hematoma in patients with MCTD is exceedingly rare and often precipitated by acute insults such as sepsis and DIC. While pre-existing vascular injury from MCTD is a central driver, the rapid evolution and severity of bleeding are frequently triggered by these systemic complications. Consequently, traditional conservative therapies are usually inadequate. Early vascular imaging—especially CTA—and timely TAE are critical for control. No standardized protocol exists, and delays can lead to catastrophic outcomes. Clinicians must therefore integrate assessment of the patient's acute infectious and coagulopathic status with their underlying autoimmune vasculopathy to guide management.

## Data Availability

The original contributions presented in the study are included in the article/Supplementary Material, further inquiries can be directed to the corresponding author.
