# Supplementary material for: Spontaneous intermuscular hematoma in mixed connective tissue disease: a case report
Source: Front Surg. 2026 Aug 19;13:1719697. doi: 10.3389/fsurg.2026.1719697 (PMC13533687; doi:10.3389/fsurg.2026.1719697)
Supplement: Supplementary file 1 [file Table1.doc]

**Supplementary materials**

**Supplementary** Table 1. Timeline Table of Major Clinical Events

| **Hospital Day** | **Corresponding Date** | **Major Clinical Event** |
| --- | --- | --- |
| Day 1 | November 9, 2024 | Admission; diagnosis of septic shock confirmed (hypotension, lactate 4.86 mmol/L, PCT >100 ng/mL); fluid resuscitation (3071 mL/24h) and norepinephrine initiated; physical examination, autoantibody panel, chest CT, and lower-limb ultrasound performed |
| Day 2 | November 10, 2024 | Overt DIC confirmed (PT 16.3 sec, INR 1.45, PTT 57.3 sec, D-dimer 99,360 μg/L FEU, PLT 56×10⁹/L); persistent thrombocytopenia documented |
| Day 3 | November 11, 2024 | Mycophenolate mofetil withheld; immunosuppressive regimen switched to IV methylprednisolone 40 mg/d |
| Day 4 | November 12, 2024 | Blood NGS sent for microbiological identification |
| Day 5 | November 13, 2024 | Blood cultures obtained; NGS and urine culture both returned *E. coli*; diagnosis of acute pyelonephritis with secondary *E. coli* bacteremia established |
| Day 8 | November 16, 2024 | Spontaneous left medial thigh hematoma (10×14 cm) developed; emergency ultrasound (Figures 2A–B) and CTA (Figure 3A) confirmed hematoma with active bleeding; arteriography performed; coil embolization of superficial femoral artery branch completed (Figures 3B–C) |
| Days 8–18 | November 16–26, 2024 | Post-embolization: hematoma stable, no expansion; lower-limb veins patent (Figures 4A–B); hemoglobin and platelets stabilized; hematoma softened |
| Day 19 | November 27, 2024 | Ischemic necrosis of hematoma bed developed (postoperative day 11); conservative management failed; surgical debridement performed (Figure 1B) |
| Discharge | (per medical records) | Patient discharged in stable condition |
